# ﻿Three new *Melanogaster* species (Boletales, Paxillaceae) from southwestern China based on morphological and molecular evidence

**DOI:** 10.3897/mycokeys.107.123565

**Published:** 2024-07-26

**Authors:** Tian-Jun Yuan, Hong-Mei Luo, Kai-Mei Su, Shu-Hong Li, Olivier Raspé

**Affiliations:** 1 School of Science, Mae Fah Luang University, Chiang Rai 57100, Thailand Mae Fah Luang University Chiang Rai Thailand; 2 Institute of Biotechnology and Germplasm Resources, Yunnan Academy of Agricultural Sciences, Kunming 650223, Yunnan, China Institute of Biotechnology and Germplasm Resources, Yunnan Academy of Agricultural Sciences Kunming China

**Keywords:** False truffles, gasteroid Boletales, phylogeny, taxonomy, three new species

## Abstract

Three newly discovered *Melanogaster* species, namely *M.cyaneus*, *M.diqingensis*, and *M.truncatisporus*, are introduced and illustrated based on both morphological and molecular data from Sichuan and Yunnan provinces in China. A multigene phylogenetic analysis (nrITS, nrLSU, and *rpb2*) was performed mainly to verify the placement of the new species in *Melanogaster*. A second, nrITS-only phylogenetic analysis comprising more *Melanogaster* species for which only ITS sequences were available, was used to infer the relationship between the new species and as many known *Melanogaster* species as possible. Specimens of *M.cyaneus*, *M.diqingensis*, and *M.truncatisporus* formed three independent clades in a phylogenetic tree inferred from the ITS data set. The robust support from ITS for these clades and genetic similarity with other species being lower than 93.2% suggest that these three species are indeed distinct from the other *Melanogaster* species in the phylogeny. Morphologically, *M.cyaneus* is characterized by its blue or bluish gleba, light brown to yellowish brown peridium, and subglobose to globose basidiospores, 6.2–15 × 4.6–9.0 μm. *Melanogasterdiqingensis* is distinguished from other *Melanogaster* species by its pale yellow to brown-yellow peridium and obovate to subglobose basidiospores, 3.0–5.1 × 2.0–4.0 μm. *Melanogastertruncatisporus* is diagnosed by its subglobose to globose or irregularly elongate-pyriform basidiomata, pale yellow to deeply orange-yellow peridium, and subglobose to globose or pyriform, truncate basidiospores. Additionally, infrageneric classification based on the number of peridium layers, the average thickness of the peridium, and the average length and width of basidiospores was tested with *M.cyaneus*, *M.diqingensis*, and *M.truncatisporus*. Orthogonal partial least squares discriminant (OPLS-DA) analysis placed the three new species within the *Melanogaster*, *Rivulares*, and *Variegati* sections, respectively. However, the morphologically circumscribed sections were not monophyletic in the phylogenetic tree. Therefore, the current infrageneric classification should be abandoned.

## ﻿Introduction

*Melanogaster* Corda, belonging to Paxillaceae within the Boletales (Basidiomycota), stands out as one of the ecologically significant groups of hypogeous fungi. According to He et al. (2019), *Melanogaster* encompasses approximately 26 species. However, recent discoveries by taxonomists worldwide have identified new species, expanding the count to 34 as listed in Index Fungorum (https://www.indexfungorum.org/, accessed on November 10, 2023). While *Melanogaster* species are predominantly distributed in the Northern Hemisphere, an exception is noted with *Melanogasterquercus* L., reported by Trappe et al. (2009) in the Southern Hemisphere. *Melanogaster* species typically establish ectomycorrhizal associations (EcM) with various plant families, including Betulaceae, Cistaceae, Fagaceae, Pinaceae, and Salicaceae (Comandini et al. 2006; Krisztián 2008; Perič and Moreau 2010; Türkoğlu and Castellano 2013; Lacheva 2015). These fungi are recognized by their peridium, varying in color from brownish to yellowish, occasionally featuring mycelial strands at the base or on the surface. The sequestrate hymenophore is composed of rounded to irregular locules of varying sizes, filled with black or dark brown basidiospores embedded in a gel and separated by whitish to yellowish veins or walls. *Melanogaster* basidiospores exhibit a range of shapes, including globose, ellipsoid, pyriform, or cirriform. Most species in the genus emit distinctive odors, ranging from sweet and pleasant to garlic-like or nauseating (Castellano et al. 1986; Montecchi and Sarasini 2000; Cázares et al. 2008; Trappe et al. 2009; de la Fuente et al. 2021; Alvarado et al. 2021; Xu et al. 2022). *Melanogaster* can be differentiated from related sequestrate Paxillaceae such as *Alpova* C.W. Dodge, *Neoalpova* Vizzini, and *Paralpova* Cabero & P. Alvarado based on differences in peridium structure. In *Melanogaster*, the peridium exhibits prostrated or interwoven hyphae, contrasting with the pseudoparenchymatous structure with inflated hyphae found in *Alpova*, *Neoalpova*, and *Paralpova*. Another false-truffle genus, *Rhizopogon* Fr. (Rhizopogonaceae), differs from *Melanogaster* by the absence of gel in the locules and the hyaline basidiospores (yellow to dark brown in *Melanogaster*). The relationships among these genera have been extensively discussed and *Melanogaster* has been placed in the Paxillinae, along with *Gyrodon* Opat., *Paxillus* Fr., and *Alpova* by Trappe (1975), Grubisha et al. (2001), Binder and Hibbett (2006), Vizzini et al. (2010), Moreau et al. (2011, 2013), and Alvarado et al. (2021).

Knapp (1954) proposed a subdivision of the genus *Melanogaster* into three groups based on the spore length (L), namely the *ambiguous* group (L > 10 μm), the *variegatus* group (7 < L < 10 μm), and the *microsporus* group (L < 7 μm). Then, Svrček (1958) proposed the subdivision into three sections, namely sect.Melanogaster (L> 10 μm), sect.Variegati (6 < L < 10 μm), and sect.Rivulares (L < 6 μm). Those sections were later validated by Moreau et al. (2011).

In China, the first specimen of *Melanogaster* was gathered from the Jinshajiang Valley in Yunnan Province, southwestern China, in 1915. Initially identified as *M.variegatus* (Vittad.) Tul. & C. Tul. by Keissler and Lohwag in 1937, it was later erected as a distinct species named *M.ovoidisporus* Y. Wang (Wang et al. 1995). Up to now, twelve species have been reported from China: *M.fusisporus* Y. Wang, *M.natsii* Y. Wang, K. Tao & B. Liu, *M.obovatisporus* B. Liu, K. Tao & Ming C. Chang, *M.ovoidisporus* Y. Wang, *M.shanxiensis* B. Liu, K. Tao & Ming C. Chang, *M.spinisporus* Y. Wang, *M.subglobisporus* K. Tao, Ming C. Chang & B. Liu, and *M.utriculatus* Y. Wang, Castellano & Trappe, *M.minobovatus*, *M.panzhihuaensis*, *M.quercicola*, *M.tomentellus* L. Fan, X. Y. Yan & Y. Y. Xu (Liu et al. 1989; Wang et al. 1995; Wang et al. 2005; Xu et al. 2022). China seems to have a rich variety of *Melanogaster* species; however, most of the previous records relied primarily on morphological evidence (Xu et al. 2022). During our survey of hypogeous fungi in Yunnan and Sichuan provinces, located in southwest China, from 2019 to 2021, we uncovered three novel species of *Melanogaster* under *Castaneamollissima* Bl. and *Quercusaquifolioides* Rehd. et Wils. Through a comprehensive analysis encompassing both morphology and phylogenetic considerations (including *Alpova*, *Neoalpova*, *Paralpova*, and *Melanogaster*), we introduce three new species: *Melanogastercyaneus*, *M.diqingensis*, and *M.truncatisporus*.

## ﻿Materials and methods

### ﻿Fungal materials

The *Melanogaster* specimens were collected from Yunnan and Sichuan Provinces in China. They were photographed in the field, placed in sterilized plastic tubes and boxes, returned to the laboratory, and stored at 4 °C. Macroscopic and microscopic descriptions were based on fresh basidiomes following the methods of Wan et al. (2016). Hand-cut sections were mounted in 5% (w/v) aqueous KOH solution, Cotton blue, or Congo red solutions and examined with an OLYMPUS BH-2 compound microscope. At least 50 basidiospores were measured of selected specimens, and the measurements are presented in the following format: L, W, Q, representing the extreme values of length, width, length to width ratio, L_m_ = L ± *S.D*., W_m_ = W ± *S.D*. and Q_m_ = Q ± *S.D*. For scanning electron microscopy (SEM), spores were scraped from the gleba of dried specimens onto double-sided tape, which was mounted directly on a SEM stub, coated with gold-palladium, examined and photographed with an SEM JSM-5600LV (JEOL, Tokyo, Japan).

The dried specimens were deposited in the Herbarium of Biotechnology and Germplasm Resources Institute of the Yunnan Academy of Agricultural Sciences (YAAS), and the Herbarium of Cryptogams, Kunming Institute of Botany, Chinese Academy of Sciences (KUN-HKAS), Yunnan, China.

### ﻿DNA extraction, PCR, and sequencing

About 10–20 mg of dried gleba were placed in a 1.5 mL tube together with one 3 mm in diameter tungsten carbide bead, and crushed by shaking two to four times for 50 s at 30Hz with a Mixer Mill MM301 (Haan, Germany). Total DNA was extracted using the CTAB method described by Hofstetter et al. (2002). The following primer pairs were used for PCR amplification: the primer pair ITS4/ITS5 was used to amplify the nuclear ribosomal internal transcribed spacer region (White et al. 1990), the primers bRPB2-5F/bRPB2-7.1R for the second largest subunit of RNA polymerase II gene (RPB2) (Matheny 2005; Matheny et al. 2007), and the LR0R/LR5 primers (Vilgalys and Hester 1990; Cubeta et al. 1991) for the 28S nrDNA region (nrLSU). Amplifications were carried out in 25 μL reaction containing 12.5 μL 2×Taq Plus Master Mix II (Vazyme Biotech Co. Ltd, China), 9.5 μL ddH_2_O, 1 μL 10 μM of forward and reverse primers, and 1 μL template DNA. Standard cycles of denaturation at 94 °C for 45 seconds, annealing for 45 seconds at different temperatures depending on the primer set (50 °C for RPB2, 54 °C for nrLSU, and 55 °C for ITS), and elongation at 72 °C for 1.5 min, followed by a final elongation step at 72 °C for 10 min. Post-cycling, samples were held at 4 °C. PCR products were sent to Shanghai Sangon Biotechnology (Shanghai, China) for purification and sequencing.

### ﻿Phylogenetic analyses

**Figure 1.**
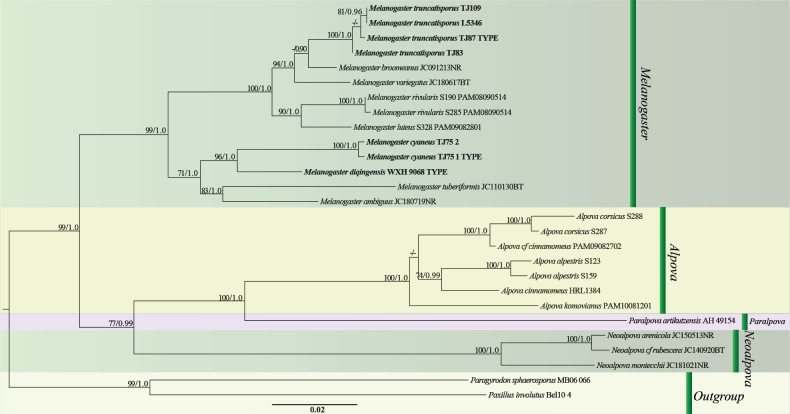
The phylogram of *Melanogaster* and closely related genera obtained with RAxML (including ITS1, ITS2, 5.8S, nrLSU and RPB2). *Paragyrodonsphaerosporus* MB06 066 and *Paxillusinvolutus* Bel10 10 were selected as the outgroup. Nodes were annotated with ML BS > 70%, Bayesian PP > 0.90, or “-” in case of non-significant support value. New species are in bold font.

The newly generated sequences were edited and assembled using SeqMan II (SeqMan Pro, DNAStar) with generic-level identifications for sequences corroborated via BLAST queries of GenBank. A total of 89 sequences (including ITS1-2, 5.8S, nrLSU, and RPB2) were used in the molecular phylogenetic analyses (Table 1), including 20 sequences newly generated in this study and 82 downloaded from GenBank. *Paragyrodonsphaerosporus* and *Paxillusinvolutus* were selected as outgroup for the multilocus phylogenetic analyses while *Alpovaalpestris*, *Alpovaconcolor*, and *Alpovacinnamomeus* were selected as outgroup for the ITS analysis. A sequence (AJ555527) of *M.tuberiformis* served as the reference for delineating 5.8S, ITS1, and ITS2. The ITS, 5.8S, nrLSU and RPB2 sequences were separately aligned using MAFFT ver. 7 (Katoh and Standley 2013) on the online server accessed at https://mafft.cbrc.jp/alignment/server/, with the G-INS-I algorithm. The obtained alignment was manually refined in BioEdit, and ambiguously aligned sites were pinpointed using Gblocks 0.91b (Castresana 2000), using default options, except “Allowed Gap Positions” = half. After Gblocks, 90%, 99%, 99.5%, and 99% of the positions were kept for ITS1-2, 5.8S, nrLSU, and RPB2, respectively. The ITS1-2, 5.8S, nrLSU, and RPB2 alignments were 537, 157, 886, 715 bp long, respectively (including gaps). The alignments were deposited in Figshare (doi: 10.6084/m9.figshare.25440544). Phylogenetic analyses were performed using maximum likelihood (ML) and Bayesian inference (BI) to validate the affiliation of our specimens with *Melanogaster*, relying on multi-gene sequences, which included *Alpova*, *Paralpova*, and *Neoalpova* (Fig. 1). Additionally, an ITS dataset (including 159 bp 5.8S, and 527 bp ITS1+ITS2) was compiled to infer the phylogenetic relationships between *Melanogaster* species. The ML analyses were performed with RAxML 8.0.14 (Stamatakis et al. 2005; Stamatakis 2014) with all parameters at default settings, except a mixed-model partitioning (with the same character sets as in the BI analyses), GTRGAMMA+I for all character sets, and 1,000 bootstrap pseudoreplicates. Partitioned BI analyses were performed with MrBayes v.3.1.2 (Ronquist and Huelsenbeck 2003) based on the following best-fit substitution models estimated by jModelTest2 on Cipres XSEDE (2.1.6, https://www.phylo.org; Miller et al. 2010). For the multi-gene dataset, 5.8S: K80+G; ITS1-2: HKY+I+G; nrLSU and RPB2: GTR+I+G). For the ITS-only dataset, 5.8S: HKY+I+G; and ITS1-2: GTR+I+G. Two independent runs of four chains were conducted for 2.5 10^6^ generations (ITS dataset) and 5 10^6^ generations (multiple-gene dataset), with trees sampled every 100 generations. The average standard deviation of split frequency (ASDSF) values at the conclusion of the runs were 0.009248 for the multi-gene tree and 0.001889 for the ITS tree. After discarding the samples from the burn-in phase (first 25% of trees), a 70% majority-rule consensus tree was constructed and posterior probabilities computed. No outgroup was specified when running the BI analyses, but the obtained tree was rerooted with the outgroups used in the ML analysis.

The trees were visualized with TreeView32 (Page 2001), exported in PDF format, and edited in Adobe Illustrator CS6. Clades with bootstrap support (BS) ≥ 70% and Bayesian posterior probabilities (PP) ≥ 0.90 were considered significantly supported (Alfaro et al. 2003).

**Table 1.** Specimen information and GenBank accession numbers for sequences used in this study.

Species	Isolate/ Strain/	Country	Genbank accession numbers			Reference
	Clone/Voucher		ITS	nrLSU	*RPB2*	
*Alpovaalpestris*	S123	France	HQ714711	/	HQ714846	Moreau et al. 2013
*Alpovaalpestris*	S159	France	HQ714721	/	HQ714853	Moreau et al. 2013
*Alpovacf.cinnamomeus*	PAM09082702	France	HQ714779	/	HQ714901	Moreau et al. 2013
*Alpovacinnamomeus*	BROWN FP73	USA	KF835996	/	/	Hayward et al. 2014
*Alpovacinnamomeus*	HRL1384	Canada	MN594282	MN594298	MN594770	Unpublished
*Alpovaconcolor*	UBC F14673	USA	KF835997	/	/	Hayward et al. 2014
*Alpovaconcolor*	OSC 65696	USA	NR_154686	/	/	Unpublished
*Alpovacorsicus*	S287	France	HQ714769	/	HQ714893	Hayward et al. 2014
*Alpovacorsicus*	S288	France	HQ714770	/	HQ714894	Moreau et al. 2011
*Alpovakomovianus*	PAM10081201	Montenegro	JQ436850	/	JQ436862	Moreau et al. 2013
*Neoalpovaarenicola*	JC150513NR	Spain	MN594292	MN594304	MN594775	Unpublished
*Neoalpovacf.rubescens*	JC140920BT	Spain	MN594294	MN594305	MN594776	Unpublished
*Neoalpovamontecchii*	JC181021NR	Spain	MN594296	MN594306	MN594777	Alvarado et al. 2021
*Paralpovaartikutzensis*	AH 49154	Spain	NR_173892	MN594307	MN594778	Alvarado et al. 2021
*Melanogasterambiguus*	B-2220	Hungary	AJ555510	/	/	Unpublished
*Melanogasterambiguus*	51745	Hungary	AJ555511	/	/	Unpublished
*Melanogasterambiguus*	B-1599	Hungary	AJ555512	/	/	Unpublished
*Melanogasterambiguus*	B-1613	Hungary	AJ555513	/	/	Unpublished
*Melanogasterambiguus*	B-2409	Hungary	AJ555514	/	/	Unpublished
*Melanogasterambiguus*	Ch12	Poland	KX438335	/	/	Unpublished
*Melanogasterambiguus*	JC180719NR	Spain	MN594286	MN594299	MN594771	Unpublished
*Melanogasterambiguus*	OSC158337 MES304	Poland	MN984308	/	/	Unpublished
*Melanogasterambiguus*	MTH1	Germany	MN994353	/	/	Unpublished
*Melanogasterbroomeanus*	JC091213NR	Spain	MN594287	MN594300	MN594772	Unpublished
*Melanogasterbroomeanus*	OTU_718s	United Kingdom	MT095837	/	/	Arraiano-Castilho et al. 2021
*Melanogasterbroomeanus*	OTU_719s	United Kingdom	MT095838	/	/	Arraiano-Castilho et al. 2021
***Melanogastercyaneus***	**TJ75_1 (TYPE)**	**China**	**ON427476**	**ON427489**	**ON533869**	**This study**
***Melanogastercyaneus***	**TJ75_2**	**China**	**ON427477**	**ON427490**	**ON533870**	**This study**
***Melanogasterdiqingensis***	**WXH_9068 (TYPE)**	**China**	**ON427482**	**ON427495**	**ON533874**	**This study**
*Melanogastereuryspermus*	OSC158352 DS1257	USA	MN984309	/	/	Unpublished
*Melanogastereuryspermus*	OSC158364 DS1555	USA	MN984310	/	/	Unpublished
*Melanogastereuryspermus*	OSC158339 JLF1044	USA	MN984311	/	/	Unpublished
*Melanogastereuryspermus*	OSC158325 JLF1129	USA	MN984312	/	/	Unpublished
*Melanogastereuryspermus*	OSC158351 JLF1456	USA	MN984313	/	/	Unpublished
*Melanogastereuryspermus*	OSC158317 JMT22778	USA	MN984314	/	/	Unpublished
*Melanogastereuryspermus*	OSC158333 MES110	USA	MN984315	/	/	Unpublished
*Melanogasterintermedius*	B-1770	Hungary	AJ555515	/	/	Unpublished
*Melanogasterintermedius*	RBG Kew K(M)130202	England	EU784372	/	/	Brock et al. 2009
*Melanogasterintermedius*	MT48	Germany	KX168661	/	/	Unpublished
*Melanogasterluteus*	S328/PAM09082801	France	HQ714780	/	HQ714902	Moreau et al. 2011
*Melanogasterluteus*	S407/Mon06	Montenegro	HQ714794	/	/	Moreau et al. 2011
*Melanogastermacrosporus*	cI-94	USA	AJ555526	/	/	Unpublished
*Melanogastermacrosporus*	B-2254	USA	AJ555528	/	/	Unpublished
*Melanogasterminobovatus*	BJTC FAN911	China	NR_186967	/	/	Xu et al. 2022
*Melanogasternatsii*	OSC82168 JMT7491	USA	MN984331	/	/	Unpublished
*Melanogasternatsii*	OSC158336 MES297	USA	MN984332	/	/	Unpublished
*Melanogasterpanzhihuaensis*	HMAS 81915	China	NR_186968	/	/	Xu et al. 2022
*Melanogasterrivularis*	S190/PAM08090514	France	HQ714731	/	HQ714862	Moreau et al. 2011
*Melanogasterrivularis*	S285/PAM08090514	France	HQ714767	/	HQ714891	Moreau et al. 2011
*Melanogasterrivularis*	LIP PAM08090514 (TYPE)	France	NR_132848	/	/	Moreau et al. 2011
*Melanogaster* sp.	Melanog002FRA	France	KU924526	/	/	Unpublished
*Melanogaster* sp.	Melanog006FRA	France	KU924529	/	/	Unpublished
*Melanogaster* sp.	Melanog007FRA	France	KU924530	/	/	Unpublished
*Melanogaster* sp.	Melanog008FRA	France	KU924531	/	/	Unpublished
*Melanogaster* sp.	Melanog011FRA	France	KU924533	/	/	Unpublished
*Melanogaster* sp.	Melanog012FRA	France	KU924534	/	/	Unpublished
*Melanogaster* sp.	Melanog018FRA	France	KU924535	/	/	Unpublished
*Melanogaster* sp.	Melanog019FRA	France	KU924536	/	/	Unpublished
*Melanogaster* sp.	MT15	Germany	KX168646	/	/	Unpublished
*Melanogaster* sp.	MES-1003	Chile	KY462394	/	/	Brock et al. 2009
*Melanogaster* sp.	LMKR1187	United Kingdom	MF352733	/	/	Suz et al. 2017
*Melanogaster* sp.	JC110118BT	Spain	MN594288	/	/	Unpublished
*Melanogaster* sp.	OSC AHF420	France	MN984333	/	/	Unpublished
*Melanogaster* sp.	OSC158378 DS1755	USA	MN984334	/	/	Unpublished
*Melanogaster* sp.	OSC LG1042	USA	MN984335	/	/	Unpublished
*Melanogaster* sp.	MVC 753_FLAS-F-65878	Chile	MT366708	/	/	Unpublished
Melanogaster sp. OK-2022a	oka331	Turkey	OP548647	/	/	Unpublished
Melanogaster sp. OK-2022a	oka332	Turkey	OP548648	/	/	Unpublished
*Melanogasterspinisporus*	BJTC FAN1092	China	MW598537	/	/	Xu et al. 2022
*Melanogasterspinisporus*	BJTC FAN938	China	MW598546	/	/	Xu et al. 2022
*Melanogasterspinisporus*	BJTC FAN941-A	China	MW598548	/	/	Xu et al. 2022
*Melanogasterspinisporus*	BJTC FAN941-B	China	MW598549	/	/	Xu et al. 2022
*Melanogastersubglobisporus*	HMAS83329	China	MW598534	/	/	Xu et al. 2022
***Melanogastertruncatisporus***	**TJ83**	**China**	**ON427478**	**ON427491**	**ON533871**	**This study**
***Melanogastertruncatisporus***	**TJ87 (TYPE)**	**China**	**ON427479**	**ON427492**	**ON533872**	**This study**
***Melanogastertruncatisporus***	**TJ109**	**China**	**ON427480**	**ON427493**	**ON533873**	**This study**
***Melanogastertruncatisporus***	**L5346**	**China**	**ON427481**	**ON427494**	/	**This study**
*Melanogastertuberiformis*	B-1295	Romania	AJ555527	/	/	Unpublished
*Melanogastertuberiformis*	JC110130BT	Spain	MN594289	MN594302	MN594773	Unpublished
*Melanogastervariegatus*	23640	Hungary	AJ555522	/	/	Unpublished
*Melanogastervariegatus*	B-1438	Hungary	AJ555523	/	/	Unpublished
*Melanogastervariegatus*	B-1688	Hungary	AJ555524	/	/	Unpublished
*Melanogastervariegatus*	B-1225	Hungary	AJ555533	/	/	Unpublished
*Melanogastervariegatus*	B-1348	Hungary	AJ555534	/	/	Unpublished
*Melanogastervariegatus*	B-2312	Hungary	AJ555535	/	/	Unpublished
*Melanogastervariegatus*	JC180617BT	Spain	MN594290	MN594303	MN594774	Unpublished
*Uncultured Melanogaster*	MFT57	Germany	FJ403505	/	/	Pena et al. 2010
*Paragyrodonsphaerosporus*	MB06-066	USA	GU187540	GU187593	GU187803	Binder et al. 2010
*Paxillusinvolutus*	Bel10.4	France	KF261366	/	JQ436854	Jargeat et al. 2014

Note: The symbol “/” means that the sequence was not available, and sequences newly generated for this study are in bold.

### ﻿Subdivision of the genus *Melanogaster* based on morphological characteristics

In order to explore the correspondence between morphological features on the subdivision of *Melanogaster* from China (including the three new species described herein) and other parts of the world, the main morphological features (including the number of peridium layers, average thickness of peridium, average length, and width of basidiospores) were selected for statistical analysis (Table 2). Using the three sections (*Melanogaster*, *Rivulares*, and *Variegati*) as the dependent variable (*Y*), and the morphological features of the species as independent variables (*X*), we conducted a visual analysis of subdivision of different species using Orthogonal partial least squares discriminant analysis (OPLS-DA) using SIMCA 14.0.

**Table 2.** The main morphological characterization of *Melanogaster* species.

Species	Peridium			Basidiospores		Country
	layers	Min. (μm)	Max. (μm)	L_m_ (μm)	W_m_ (μm)	
*M.quercicola*	2	450	600	13.1	6.5	China
*M.utriculatus*	2	300	400	13	9	Japan
*M.fusisporus*	2	250	420	12.3	5.2	China
*M.shanxiensis*	2	180	420	12.2	6.1	China
*M.obovatus*	2	350	600	12.2	6	China
*M.macrosporus*	1	85	315	11.5	5.7	Hungary
*M.panzhihuaensis*	1	150	220	10.4	6.1	China
*M.natsii*	2	200	250	10	6	China
*M.coccolobae*	2	170	230	9.9	7.5	Mexico
*M.subglobisporus*	2	130	300	9.8	7	China
*M.spinisporus*	1	360	750	9.6	7.3	China
***M.cyaneus***	2	100	400	9.5	7	China
*M.tomentellus*	1	250	350	9.4	4.5	China
*M.ovoidisporus*	2	250	450	7.1	4.3	China
***M.truncatisporus***	2	200	450	7	4.5	China
*M.minobovatus*	2	350	500	6.1	4.7	China
*M.broomeanus*	1	150	400	6	3.9	China
*M.luteus*	2	100	250	6	2.75	France, Montenegro
*M.minysporus*	1	160	240	5.5	4	Mexico
*M.rivularis*	2	100	135	5.5	2.9	France
***M.diqingensis***	2	100	320	3.8	3.2	China

Notes: The new species introduced in this study are presented in bold.

## ﻿Results

### ﻿Molecular data analyses

**Figure 2.**
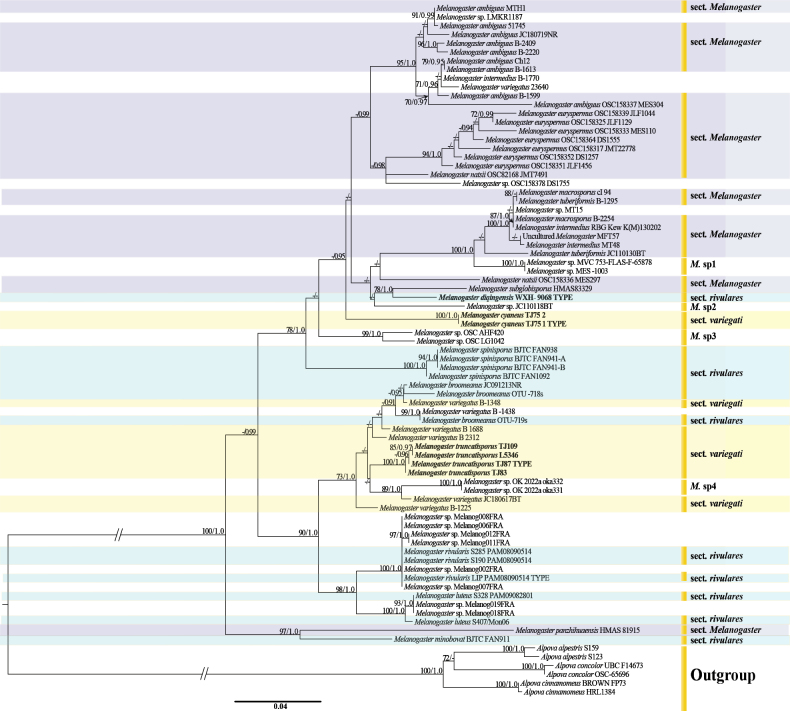
The ITS phylogram of *Melanogaster* obtained with RAxML (including ITS1, ITS2, and 5.8S). The six *Alpova* cessions were chosen as the outgroup. Nodes were annotated with ML BS > 70%, Bayesian PP > 0.90, or “-” in case of non-significant support value. New species are in bold font. The sections are as defined by Moreau et al. 2011 on the basis of basidiospore length.

The multi-gene dataset (ITS, 5.8S, nrLSU and RPB2) contained 27 specimens (7 novel specimens from our collections) and had an aligned length of 2,295 characters. ML and BI yielded identical tree topologies and only the tree inferred from the ML analysis is shown (Fig. 1). The ITS dataset contained 79 specimens, of which 7 were newly sequenced, and had an aligned length of 683 characters. ML and BI analyses produced identical tree topologies and only the tree derived from the ML analysis is shown (Fig. 2). The four-gene dataset and ITS (ITS1-2 and 5.8S) resulted in ML trees with a log-likelihood of -9919.6 and -5052.3, respectively. The general topology of the trees (Figs 1, 2) is congruent with those already published by Moreau et al. (2011, 2013). The phylogenetic tree derived from the four-gene dataset (Fig. 1), validates the classification of our specimens in *Melanogaster*. Our 7 specimens were resolved as 3 strongly supported species-level clades or branches, different from all known species included in the analysis (Fig. 2). Furthermore, two sequences (*Melanogaster* sp. MVC 753 FLASF 65878 and *Melanogaster* sp. MES 1003) from Chile, a sequence (*Melanogaster* sp. JC110118BT) from Spain, two sequences (*Melanogaster* sp. OSC AHF420 from France, *Melanogaster* sp. OSC LG1042 from the USA), and two sequences (*Melanogaster* sp. OK 2022a oka331 and *Melanogaster* sp. OK 2022a oka332) from Turkey formed four distinct, species-level clades/lineages labelled *M.* sp1 to *M.* sp4 (Fig. 2). These clades exhibited less than 98.4% similarity (*M.* sp1 = 98.3%, *M.* sp2 = 93.47%, *M.* sp3 = 94.06%, and *M.* sp4 = 96.63%) in their ITS sequences compared to other species of *Melanogaster*. This observation suggests the likely presence of four additional undescribed species.

### ﻿Taxonomy

#### ﻿ Melanogaster cyaneus

Taxon classificationFungiBoletales, Paxillaceae Paxillaceae

T. J. Yuan, Shu H. Li, & Raspé sp. nov.

B523B224-6487-50D1-B446-FD1EEFCA1849

MycoBank No: 901868

Fig. 3a–d

##### Diagnosis.

*Melanogastercyaneus* is diagnosed by its blue or bluish gleba, rhizomorphs on the base, thinner peridium (100–400 μm), and longer and wider basidiospores (6.2–15 × 4.6–9.0 μm).

##### Etymology.

The epithet *cyaneus* refers to the blue or bluish gleba.

##### Holotype.

China. Sichuan Province: Panzhihua City, Yanbian County, Shuanglong village, 26°49'12"N, 101°33'7.1028"E, elevation 1,970 m, in mainly reddish brown soils under *Castaneamollissima* Bl., 16 Aug. 2020, collected by M. Yang (KUN- HKAS129200, holotype; YAAS-TJ75-1, isotype).

##### Description.

**Figure 3.**
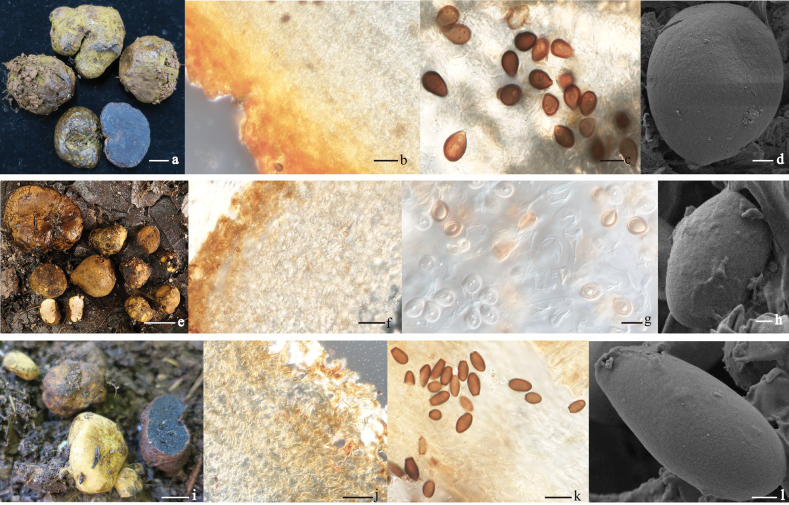
Photographs of *Melanogaster* species. *Melanogastercyaneus* (YAAS-TJ75-1, holotype) **a** basidiomata **b** LM of Peridium **c** LM of basidiospores **d** SEM of basidiospore. *Melanogasterdiqingensis* (YAAS-WXH_9068, holotype) **e** basidiomata **f** LM of Peridium **g** LM of basidiospores **h** SEM of basidiospore. *Melanogastertruncatisporus* (YAAS-TJ87, holotype) **i** basidiomata **j** LM of peridium **k** LM of basidiospores **l** SEM of ascospore. Scale bars: 2 cm (**a, e, i**); 20 μm (**b, f, g**); 10 μm (**c, g, k**); 1 μm (**d, h, l**).

***Basidiomata*** 2.5–4.0 × 1.5–2.5 cm, hypogeous or semi-hypogeous, subglobose to ellipsoidal, occasionally elongate; light brown to yellowish brown, with mycelial strands attached in the base (Fig. 3a). ***Peridium*** two-layered, outer layer 20–75 μm thick, composed of interwoven hyphae, orange-yellow to reddish, 2–4 μm broad, thick-walled, clamp connections present; inner layer 145–335 μm thick, composed of interwoven, strongly gelatinized hyphae, 2–5 μm broad, pale-yellow, intermixed with massive inflated cells, 6–12 μm broad, with clamp connections present. ***Gleba*** solid, gelatinous, milk-white when immature, blue or bluish at mature; trama plates pale-yellow, of gelatinized hyphae (Fig. 3b); locules small, filled with black spores (Fig. 3c). ***Basidia*** exhibit limited revival, appearing clavate, hyaline, 4-spores, randomly distributed, gelatinized at maturity. ***Basidiospores*** subglobose to globose, 6.2–15 × 4.6–9.0 μm (L_m_ × W_m_= 9.5 ± 3.0 × 7.0 ± 2.0, *Q*= 1.0–2.0, *Q_m_* =1.4 ± 0.6, n = 75), smooth, hyaline when immature, light yellow to reddish at maturity, surfaces display distinctive spots, and optical microscopy reveals a notable hilar appendage, 0.5–1.0 μm in diam (Fig. 3d).

##### Other material examined.

China, Sichuan province, Panzhihua City, Yanbian County, Shuanglong village, 26°49'12"N, 101°33'7.1029"E, elevation 2,000 m, under *Castaneamollissima* Bl. in evergreen hill forest, in mainly reddish-brown soils, 16 Aug. 2020, collected by M. Yang (YAAS TJ75-2).

##### Notes.

*M.broomeanus* Berk (Türkoğlu and Castellano 2013; Uzun et al. 2014), *M.shanxiensis* and *M.obovatisporus* (Liu et al. 1989), are similar to *M.cyaneus* in morphology. *M.cyaneus* (basidiomata light brown to yellowish brown), *M.broomeanus* (basidiomata yellow-brown to deep brown), *M.shanxiensis* (basidiomata brown to rust-brown) and *M.obovatisporus* (basidiomata brown to brownish-black), but the specimens of *M.cyaneus* clustered in an independent clade with strong support (BS = 100%, PP = 1.0; Fig. 2), supporting it as a distinct species. Additionally, DNA analysis revealed *M.cyaneus* shared less than 92% ITS similarity with other *Melanogaster* species.

#### ﻿ Melanogaster diqingensis

Taxon classificationFungiBoletales, Paxillaceae Paxillaceae

T. J. Yuan, Shu H. Li, & Raspé sp. nov.

17864080-6B99-5FAF-84B1-6F4D8EEA6420

MycoBank No: 901869

Fig. 3e–h

##### Diagnosis.

*Melanogasterdiqingensis* is diagnosed by the combination of medium-sized, pale-yellow to orange or brown-yellow basidiomata with scaled, lobed or concave surface, pale yellow gleba, and obovate to subglobose, smooth basidiospores (3.0–5.1 × 2.0–4.0 μm, *Q* = 1.0–1.8).

##### Etymology.

The epithet *diqingensis* refers to the prefecture of the type locality.

##### Holotype.

China. Yunnan Province: Diqing Autonomous Prefecture, Shangri-La County, Baishuitai village, 27°30'14.2236"N, 100°2'50.5716"E, elevation 2,380 m, in brown soil under *Quercusaquifolioides* Rehd. et Wils., 25 Sep. 2020, collected by X. H. Wang (KUN- HKAS121212, holotype; YAAS-WXH_9068, isotype).

##### Description.

***Basidiomata*** 0.5–2.5 × 0.2–2.2 cm, hypogeous, globose, subglobose or ellipsoidal, pale-yellow to orange or brown-yellow, scaled, lobed or concave in the surface, without visible rhizomorphs (Fig. 3e). ***Odor*** faint. ***Peridium*** two-layered, outer layer 40–80 μm thick, composed of interwoven hyphae, 3.5–7.5 μm broad, with fusoid to cylindrical terminal cells, deeply orange-yellow walls toward surface, with clamp connections; inner layer 150–260 μm thick, composed of interwoven hyphae, 5–10 μm broad, with inflated cells, 7.5–15 μm broad, bright pale yellow, with abundant clamp connections. ***Gleba*** solid, milk-white when immature, pale yellow at maturity, hard when dried; trama plates of hyaline or yellowish, gelatinized hyphae (Fig. 3f); locules 2–3 mm in diam (Fig. 3g). ***Basidia*** exhibit limited reviving, appearing clavate, hyaline, 4–spored. ***Basidiospores*** obovate to subglobose, smooth, 3.0–5.1 × 2.0–4.0 μm (L_m_ × W_m_= 3.8 ± 1.0 × 3.2 ± 0.8, *Q*= 1.0–1.8, *Q_m_* =1.2 ± 0.6, n = 75), hyaline (immature) to light yellow, dark brown (mature) in KOH 5%, with truncate-cupped base and very short hilar appendage in optical microscopy, 0.5–1.5 μm in diam (Fig. 3h).

##### Notes.

Six *Melanogaster* species, namely *M.subglobisporus*, *M.natsii*, *M.spinisporus* (Wang et al. 1995), *M.rivularis*, *M.luteus=M. microsporus* (Moreau et al. 2011), are similar to *M.diqingensis* in morphology and related by phylogeny. The colors of their basidiomata are similar to *M.diqingensis*, i.e. rust brown to deep brown in *M.subglobisporus*, yellow-brown in *M.natsii*, grayish brown to light brown in *M.spinisporus*, and bright golden yellow in *M.minysporus*, but the basidiomata of all of latter species are without scales, lobes or concave area, by which they were easily differentiated from *M.diqingensis*. *M.rivularis* (anthracite-black gleba), and *M.luteus* (club-shaped or cylindro-elliptical to cylindrical basidiospores) also are easily differentiated from *M.diqingensis*. The thickness of the peridium (≤ 300 μm) is similar among *M.subglobisporus*, *M.ovoidisporus*, and *M.coccolobae*, However, the size of basidiospores provide a clear distinction between these species (*M.subglobisporus* 8–11 × 7–9 μm, *M.ovoidisporus* 5–7 × 3.5–5.8 μm and *M.coccolobae* 6.2–12 × 5.2–10 μm). Phylogenetically, *M.diqingensis* and *M.subglobisporus* formed an independent and strongly supported clade (BS = 78%, PP = 1.0; Fig. 2), but *M.diqingensis* shared less than 93.2% ITS similarity with *M.subglobisporus*, supporting *M.diqingensis* as a distinct species.

#### ﻿ Melanogaster truncatisporus

Taxon classificationFungiBoletales, Paxillaceae Paxillaceae

T. J. Yuan, Shu H. Li, & Raspé sp. nov.

E59F3129-9164-52F0-B449-D10219E9C4BD

MycoBank No: 901870

Fig. 3i–l

##### Diagnosis.

*Melanogastertruncatisporus* is diagnosed by the combination of medium-sized basidiomata with orange-yellow peridium that becomes reddish brown to dark brown in age, and truncate basidiospores.

##### Etymology.

The epithet *truncatisporus* refers to the truncate basidiospores.

##### Holotype.

China. Yunnan Province: Nujiang Autonomous Prefecture, Lanping County, Zhongpai township, Xinchangping village, 26°54'15"N, 99°10'32"E, elevation 1990 m, in mainly brown soils under *Castaneamollissima* and *Pinusyunnanensis* Franch., 26 Oct. 2020, collected by T. J. Yuan (KUN-HKAS129199, holotype; YAAS-TJ87, isotype).

##### Description.

***Basidiomata*** 1.5–3.0 × 0.4–2.3 cm, hypogeous or semi-hypogeous, subglobose to oval, occasionally irregular-elongated, yellowish when young, reddish brown to dark brown at maturity, smooth or slightly velvety surface, lobed or indented at the base, attached mycelial strands, occasionally extending to the surface, dark brown, rhizomorphs not distinct (Fig. 3i). ***Peridium*** two-layered, outer layer 50–100 μm thick, composed of interwoven hyphae, orange-yellow, with clamp connections, and fusoid to cylindrical terminal cells, 4–5 μm broad; inner layer 150–350 μm thick, composed of interwoven hyphae, 3–5 μm broad, pale yellow, intermixed with massive inflated cells, ellipsoidal or irregular, 3–20 μm broad. ***Gleba*** solid, pale brown when young, blackish brown to black at maturity, separated by white or pale yellow trama when young, which becomes deep brown at maturity, hard when dried; trama plates of hyaline or yellowish gelatinized hyphae (Fig. 3j); locules 2–4 mm in diameter, polygonal to irregular (Fig. 3k). ***Basidia*** poorly recovered, hyaline, 4–spored, occurring randomly in the locules (Fig. 3k). ***Basidiospores*** subglobose to globose or irregularly elongate-pyriform, 3.5–9.5 × 3.0–7.0 μm (L_m_ × W_m_ = 7.0 ± 2.5 × 4.5 ± 2.0, *Q* = 1.0–2.5, *Q_m_* = 1.5 ± 1.0, n = 65), hyaline when immature, becoming dark brown at maturity, smooth, with truncate-cupped base and short hilar appendage,1–2 μm in diam in optical microscopy (Fig. 3l).

##### Habitat, phenology, and distribution.

hypogeous to semi-hypogeous under *Castaneamollissima* and *Pinusyunnanensis*, in mixed forest, in late autumn. So far found in Lanping and Gongshan counties, Yunnan Province, China.

##### Other material examined.

China. Yunnan Province: Nujiang autonomous Prefecture, Lanping County, 26°54'17"N, 99°10'31"E, elevation 2,030 m, in mainly brown soils under *Castaneamollissima* and *Pinusyunnanensis*, 26 Oct. 2020, collected by T. J. Yuan (YAAS TJ83 and YAAS TJ109); China. Yunnan Province: Nujiang Autonomous Prefecture, Gongshan County, 28°1'19"N, 98°37'2"E, elevation 1,800 m, in mainly brown soils under *Castaneamollissima* and *Pinusyunnanensis*, 25 Sep. 2020, collected by Li, S. H. (YAAS L5346).

##### Notes.

Four *Melanogaster* species, namely *M.minysporus* (Cázares et al. 2008), *M.broomeanus* Berk (Türkoğlu and Castellano 2013, Uzun et al. 2014), *M.obovatisporus* (Liu et al. 1989), and *M.variegatus* (Krisztián 2008), are similar in morphology and related to *M.truncatisporus* by phylogeny. *M.truncatisporus* can be easily differentiated by its peridium thickness (*M.truncatisporus*, 200–450 μm vs *M.minysporus*, 160–240 μm) and the size of basidiospores (*M.truncatisporus*, 3.5–9.5 × 3.0–7.0 μm vs *M.minysporus*, 5–6.5 × 3–5 μm). *M.truncatisporus* has a two-layered peridium and *M.broomeanus* has a single-layered peridium. The difference in basidiospore size is evident, with 3.5–9.5 × 3.0–7.0 μm for *M.truncatisporus* and 7–9 × 4 μm for *M.broomeanus*. Also, *M.truncatisporus* basidia typically contain 4 spores, whereas those of *M.obovatisporus* consistently contain 8 spores. Basidiospore size (especially minimum size) is also a diagnostic character to separate *M.truncatisporus* from *M.variegatus* (3.5–9.5 × 3.0–7.0 μm for the former, and 7.5–10 × 5.5–8 μm for the latter). Phylogenetically, the specimens of *M.truncatisporus* clustered in an independent clade with strong support (BS = 100%, PP = 1.0; Fig. 2), supporting it as a new species. Additionally, *M.truncatisporus* (holotype ITS sequence ON427479) shared less than 93.2% similarity with ITS sequences of other *Melanogaster* species.

### ﻿Subdivision of the genus *Melanogaster* in sections based on morphology

**Figure 4.**
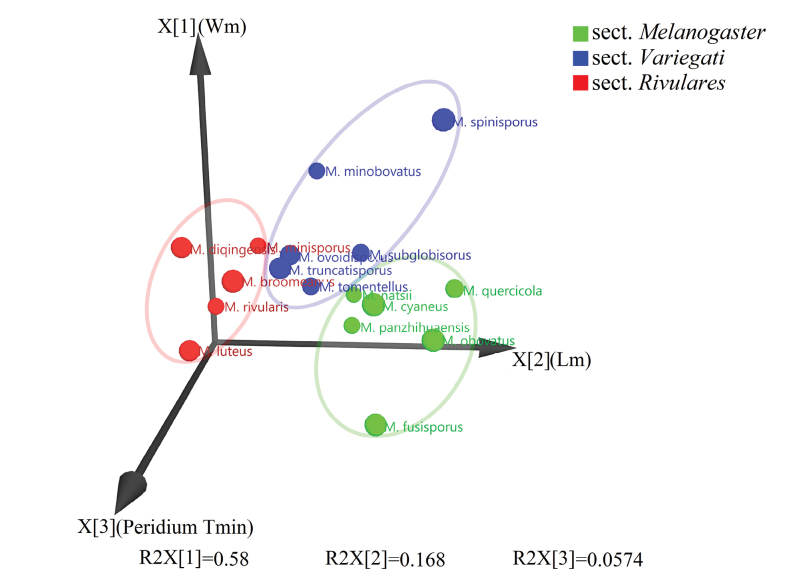
OPLS-DA discriminant analysis of three sections including Chinese species, based on their main morphological characteristics. Lm = mean spore length; Wm = mean spore width; Peridium Tmin = minimum thickness of peridium.

The main characters to identify *Melanogaster* species are the number of layers of the peridium, thickness of the peridium, and average length and width of basidiospores (Table 2). A discriminant analysis based on five variables, namely the number of layers of the peridium, the minimum and maximum thickness of the peridium, and the length and width of basidiospores, was performed by OPLS-DA (Fig. 4). In this analysis, the three sections, *Melanogaster*, *Variegati*, and *Rivulares*, were separated in the 3D principal component space by the average length and average width of basidiospores, and the minimum thickness of the peridium. The cumulative contribution of the first three principal components exceeded 80.5%. The primary factors determining the distribution of species in the 3-dimensional space are the average length and width of spores. The three new species (*M.cyaneus*, *M.diqingensis*, and *M.truncatisporus*) were placed into the sectionMelanogaster, *Rivulares*, and *Variegati* by OPLS-DA, respectively.

## ﻿Discussion

In this paper, we introduce three novel species of hypogeous fungi belonging to *Melanogaster* (Paxillaceae, Boletales): *M.cyaneus*, *M.diqingensis*, and *M.truncatisporus*. The first two species were discovered in Yunnan Province, while the latter was collected in Sichuan Province, in southwest China. Morphologically, *M.cyaneus* stands out with its distinctive blue or bluish gleba and light brown to yellowish brown basidiomata. *M.diqingensis* can be identified by its pale yellow to brown-yellow basidiomata, featuring a scaled, lobed, or concave surface. *M.truncatisporus* is characterized by its distinct pale yellow to deeply orange-yellow peridium and subglobose to globose or pyriform basidiospores. These characteristics differentiate them from other *Melanogaster* species found in China. Phylogenetically, our analysis based on an ITS rDNA dataset, including ITS1-2 and 5.8S, revealed that specimens of *M.cyaneus* and *M.truncatisporus* clustered into two independent clades with strong support (BS = 100%, PP = 1.0). *M.diqingensis* and *M.subglobisporus* formed a separate and well-supported clade (BS = 78%, PP = 1.0; Fig. 2). Moreover, these three species shared less than 93.2% ITS similarity with other *Melanogaster* species. Both the phylogenetic tree and morphological features concur to support these three species as new additions to *Melanogaster*.

Knapp (1954) and Moreau et al. (2011) proposed a division of European *Melanogaster* species into three sections based on the average length of basidiospores. These sections were defined as follows: sectionMelanogaster, with basidiospores longer than 10 μm; sectionVariegati, with basidiospores measuring 6–10 μm; and sectionRivulares, with basidiospores ranging from 5 to 6 μm. Our multivariate statistical analysis based on wider taxon sampling and three morphological characters instead of only spore length allowed to separate species in line with Moreau et al. (2011) sections (Fig. 4). However, in the phylogenetic tree (Fig. 2), the three morphologically delineated sections were not monophyletic. For example, *M.panzhihuaensis* described from China was placed within the *Melanogaster* section based on morphology (Fig. 4), but shared a sister relationship with *M.minobovatus*, also from China, which was assigned to sectionRivulares by the multivariate morphological analysis (Fig. 4). This placement is distinct from other species classified within the *Melanogaster* section, such as *M.ambiguus* (Spain and China), *M.intermedius* (UK), *M.euryspermus* (USA), *M.tuberiformis* (Spain), *M.natsii* (China), *M.subglobisporus* (China), and *M.macrosporus* (Hungary) (Wang et al. 1995; Frank et al. 2006; Brock et al. 2009; Alvarado et al. 2021; Xu et al. 2022).

A set of 19 *Melanogaster* sequences from Germany, France, and the USA (as detailed in Table 1) were included in our phylogenetic analyses. The phylogenetic position of certain specimens based on these sequences was problematic, particularly concerning *M.intermedius* B-1770, and *M.variegatus* 23640 and B-1688 from Hungary, as well as *M.natsii* OSC82168 JMT7491 and OSC158336 MES297 from the USA. All those accessions belonged in species-level clades including other accessions identified as different species. The ITS rDNA sequences of *Melanogaster* sp. (Melanog002FRA, Melanog006FRA, Melanog007FRA, Melanog008FRA, Melanog011FRA, and Melanog012FRA), along with *Melanogaster* sp. (Melanog018FRA and Melanog019FRA) from France, clustered with *M.rivularis* and *M.luteus* clades, respectively. This observation is substantiated by robust support from high bootstrap values (BS = 97 and BS = 93) and posterior probability from Bayesian inference (PP = 1.0) in Fig. 2. Consequently, we recommend classifying the first six unidentified specimens as *M.rivularis* species and the last two specimens as *M.luteus*.

*Melanogaster* sp. MVC 753 FLASF 65878 (Chile) and *Melanogaster* sp. MES 1003 (Chile) exhibited less than 98.3% similarity in the ITS region when compared to *M.macrosporus*. Furthermore, an ITS sequence (*Melanogaster* sp. JC110118BT) from Spain, two ITS sequences (*Melanogaster* sp. OSC AHF420 from France and *Melanogaster* sp. OSC LG1042 from the USA), and two ITS sequences (*Melanogaster* sp. OK 2022a oka331 and *Melanogaster* sp. OK 2022a oka332) from Turkey exhibited less than 96.7% similarity to ITS sequences of other *Melanogaster* species. Consequently, those accessions represent four distinct species-level lineages, designated as *M.* sp1 to *M.* sp4, respectively (Fig. 2). This observation suggests the presence of four undescribed species. More research is therefore needed to validate and properly describe those putative novel species.

### ﻿Key to the *Melanogaster* species from China

**Table:** 

1a	Basidiospores verrucose	**2**
1b	Basidiospores smooth	**3**
2a	Basidiomata brown to deep brown, peridium 250–350 μm thick, surface minutely green tomentose; basidiospores elongate fusiform	***M.tomentellus***
2b	Basidiomata light brown with some red-brown or brick-red when fresh, peridium 360–750 μm thick, basidiospores ellipsoid to obovoid, subglobose	***M.spinisporus***
3a	Basidiospores light yellow or yellow	**4**
3b	Basidiospores brown to dark brown or reddish brown	**5**
4a	Basidiomata brick-red with dark green to black-green gleba at maturity	***M.obovatus***
4b	Basidiomata red-brown to brown with yellow-green gleba at maturity	***M.quercicola***
5a	Basidiospores mostly longer than 10 μm	**6**
5b	Basidiospores mostly shorter than 10 μm	**7**
6a	Basidiomata yellow-brown to brown, gleba brown, basidiospores fusiform	***M.fusisporus***
6b	Basidiomata brown to rust-brown, gleba brownish, basidiospores long obovoid	***M.shanxiensis***
6c	Basidiomata light brown to yellowish brown, gleba bluish, basidiospores subglobose to globose	***M.cyaneus***
6d	Basidiomata yellow-brown, gleba brown to dark brown, basidiospores limoniform or ellipsoid	***M.natsii***
7a	Basidiospores oblong to cylindrical	***M.broomeanus***
7b	Basidiospores obovate or obovate to subglobose	**8**
7c	Basidiospores subglobose to globose	**9**
8a	Basidiospores red-brown, 8.7–12.4 × 5.2–7.6 µm	***M.panzhihuaensis***
8b	Basidiospores dark brown to brownish, 6.5–8 × 3.8–4.7 µm	***M.obovatisporus***
8c	Basidiospores yellowish brown to brown, 8.7–10.6 × 6.6–8.0 µm	***M.subglobisporus***
8d	Basidiospores dark brown, 5.1–6.9 × 4.2–5.1 μm	***M.minobovatus***
9a	Basidiomata reddish brown to dark brown, gleba brownish-black or black, basidiospore 3.5–9.5 × 3.0–7.0 μm	***M.truncatisporus***
9b	Basidiomata pale yellow to orange, gleba pale yellow, basidiospore 3.0–5.1 × 2.0–4.0 μm	***M.diqingensis***

## Supplementary Material

XML Treatment for Melanogaster cyaneus

XML Treatment for Melanogaster diqingensis

XML Treatment for Melanogaster truncatisporus

## ﻿Additional information

### Author ORCIDs

Olivier Raspé https://orcid.org/0000-0002-8426-2133

### Data availability

All of the data that support the findings of this study are available in the main text, or in publicly accessible repositories as indicated in the text.
